# The link between hearing impairment and child maltreatment among Aboriginal children in the Northern Territory of Australia: is there an opportunity for a public health approach in child protection?

**DOI:** 10.1186/s12889-020-8456-8

**Published:** 2020-04-06

**Authors:** Vincent Yaofeng He, Steven Guthridge, Jiunn-Yih Su, Damien Howard, Kylie Stothers, Amanda Leach

**Affiliations:** 1 Menzies School of Health Research, Charles Darwin University, Casuarina, Northern Territory 0811 Australia; 2 Phoenix Consulting, Nightcliff, Northern Territory 0810 Australia; 3 Indigenous Allied Health Australia, Katherine, Northern Territory 0850 Australia

**Keywords:** Child maltreatment, Child abuse and neglect, Hearing impairment, Hearing loss, Data-linkage, Aboriginal children, Remote communities, Indigenous health

## Abstract

**Background:**

International studies provide evidence of an association between child disabilities, including hearing impairment (HI), and child maltreatment. There are high prevalences of ear disease with associated HI, and child maltreatment among Australian Aboriginal children, but the link between HI and child maltreatment is unknown. This study investigates the association between HI and child maltreatment for Aboriginal children living in the Northern Territory (NT) of Australia.

**Methods:**

This was a retrospective cohort study of 3895 Aboriginal school-aged children (born between 1999 and 2008) living in remote NT communities. The study used linked individual-level information from health, education and child protection services. The outcome variables were child maltreatment notifications and substantiations. The key explanatory variable, HI, was based on audiometric assessment. The Kaplan–Meier estimator method was used in univariate analysis; Cox proportional hazards regression was used in multivariable analysis.

**Results:**

A majority of the study cohort lived in very remote (94.5%) and most disadvantaged (93.1%) regions. Among all children in the study cohort, 56.1% had a record of either HI or unilateral hearing loss (UHL), and for those with a history of contact with child protection services (*n* = 2757), 56.7% had a record of HI/UHL (*n* = 1564). In the 1999–2003 birth cohort, by age 12 years, 53.5% of children with a record of moderate or worse HI had at least one maltreatment notification, compared to 47.3% of children with normal hearing. In the 2004–2008 cohort, the corresponding results were 83.4 and 71.7% respectively. In multivariable analysis, using the full cohort, children with moderate or worse HI had higher risk of any child maltreatment notification (adjusted Hazard Ratios (adjHR): 1.16, 95% CI:1.04–1.30), notification for neglect (adjHR:1.17, 95% CI:1.04–1.31) and substantiation (adjHR:1.20, 95% CI:1.04–1.40), than children with normal hearing. In the 2004–2008 birth cohort, children with moderate or worse HI had higher risk of a substantiated episode of physical abuse (adjHR:1.47, 95% CI:1.07–2.03) than children with normal hearing.

**Conclusion:**

Our findings demonstrate the urgent need for HI and child maltreatment prevention strategies through raised community awareness and inter-agency collaboration. Effective information-sharing between service providers is a critical first step to a public health approach in child protection.

## Background

International studies report an increased risk of maltreatment for children with disabilities [1–4], including children with hearing impairment (HI) [3–5]. Studies with hearing impaired children suggest that difficulties with communication may result in frustration and stress in parent-child relationships, leading to increased use of physical discipline [3, 6–9]. However studies of the association between hearing impairment and child maltreatment [3–5] do not differentiate between sensorineural and conductive HI [10] and may be missing more nuanced strategies to prevent child maltreatment. Sensorineural hearing loss in children may be genetic, or the result of accident, toxins or infections and is not readily treated [11]. One common strategy to improve communication is the use of sign language. By contrast, conductive hearing loss in children is commonly caused by middle ear infections (otitis media (OM)) or allergies and is generally considered to be preventable or correctable with surgery [12]. Conductive hearing loss is generally less severe than sensorineural hearing loss, may fluctuate over time and is less readily identifiable. To the best of our knowledge, there have been no studies that have specifically investigated the association between conductive hearing loss and child maltreatment.

Similar to Aboriginal and Torres Strait Islander populations elsewhere in Australia and First Nations populations in other countries, many Aboriginal people in the NT suffer significant disadvantage, including poverty, poor education, overcrowded living conditions and ill health [13–16]. In remote NT communities, OM and other respiratory infections are common among Aboriginal infants and young children [17, 18], with reported prevalence of OM as high as 91% [17]. In this population, OM is commonly asymptomatic [19, 20], develops early in life with peak prevalence reported at 5–9 months [20], and persists through childhood. If left untreated or not treated adequately, OM often results in persistent mild to moderate conductive hearing loss, which reduces children’s exposure to language and delays language learning development [21, 22]. Poverty and overcrowding are not only associated with the high prevalence of OM and other respiratory infections amongst NT Aboriginal children [23], they may also contribute to the over-representation of Aboriginal children coming into contact with child protection services (CPS), a relationship supported by international studies that have demonstrated a link between neighbourhood overcrowding and child maltreatment [24, 25]. In the NT, Aboriginal children make up 43% of all NT children [26, 27] but are 82% of NT children in contact with the CPS [28]. This disparity is evident in the high rates of NT Aboriginal children who receive child protection services (225.7 per 1000 children), compared to all children across Australia (28.7 per 1000 children) [28].

Despite the high prevalence of OM among NT Aboriginal children, OM often remains undetected. While OM is reported from research studies to develop early in life [20], it is frequently not diagnosed in clinical practice until an older age when children are more easily examined [17]. As suggested in a previous NT study, parents may also be unaware of their child’s ear infections and related hearing loss with the result that behaviour of children with hearing loss may be interpreted by families as defiant and disrespectful and children may be punished for wilfully defying instructions when in fact they have not heard what was said to them [29]. Children with hearing loss may also become frustrated when they do not understand what is said or are unable to make their wants understood, leading to aggression towards caregivers and peers [29]. Both of these responses may, especially if occurring regularly and/or escalating, may come to the attention of CPS.

The current literature on the HI-maltreatment link has focussed on children with sensorineural hearing loss and in urban settings, with a lack of studies that examine such link for conductive hearing loss or in remote settings. Previous research has investigated the effects of hearing loss on family life and suggested mechanisms by which hearing loss may contribute to child protection reports in remote communities [29], but there is a lack of empirical studies that provide a comprehensive whole-of-population perspective on the risk of contact with the CPS for children with varying levels of HI. In addition, there are limited studies that explore the impact of HI on the different maltreatment types – neglect, physical, emotional and sexual abuse. This is particularly relevant to the NT which has a different pattern of child protection reporting compared to Australia as a whole. In the NT, the leading type of substantiated maltreatment is neglect (45.9%), followed by emotional abuse (37.2%), physical abuse (14.9%) and sexual abuse (2.0%), while across Australia the most common type of substantiated maltreatment is emotional abuse (58.8%), followed by neglect (17.1%), physical abuse (14.8%) and sexual abuse (8.9%) [28]. One consequence of the different distribution of child maltreatment types, especially the high proportion of neglect, is the results of studies elsewhere in Australia and in other countries might not be generalisable to the NT. In the last decade, there has been growing acknowledgement of the need for a public health approach to address the underlying causes of child maltreatment, including the requirement for interagency collaboration to address poor health and social outcomes of children [30–33].

To address current gaps in knowledge and to inform a public health approach to child protection, the aim of this study is to investigate the association between OM-related HI and child maltreatment for young Aboriginal children living in remote NT communities.

## Methods

### Study design and study cohort

**Fig. 1:**
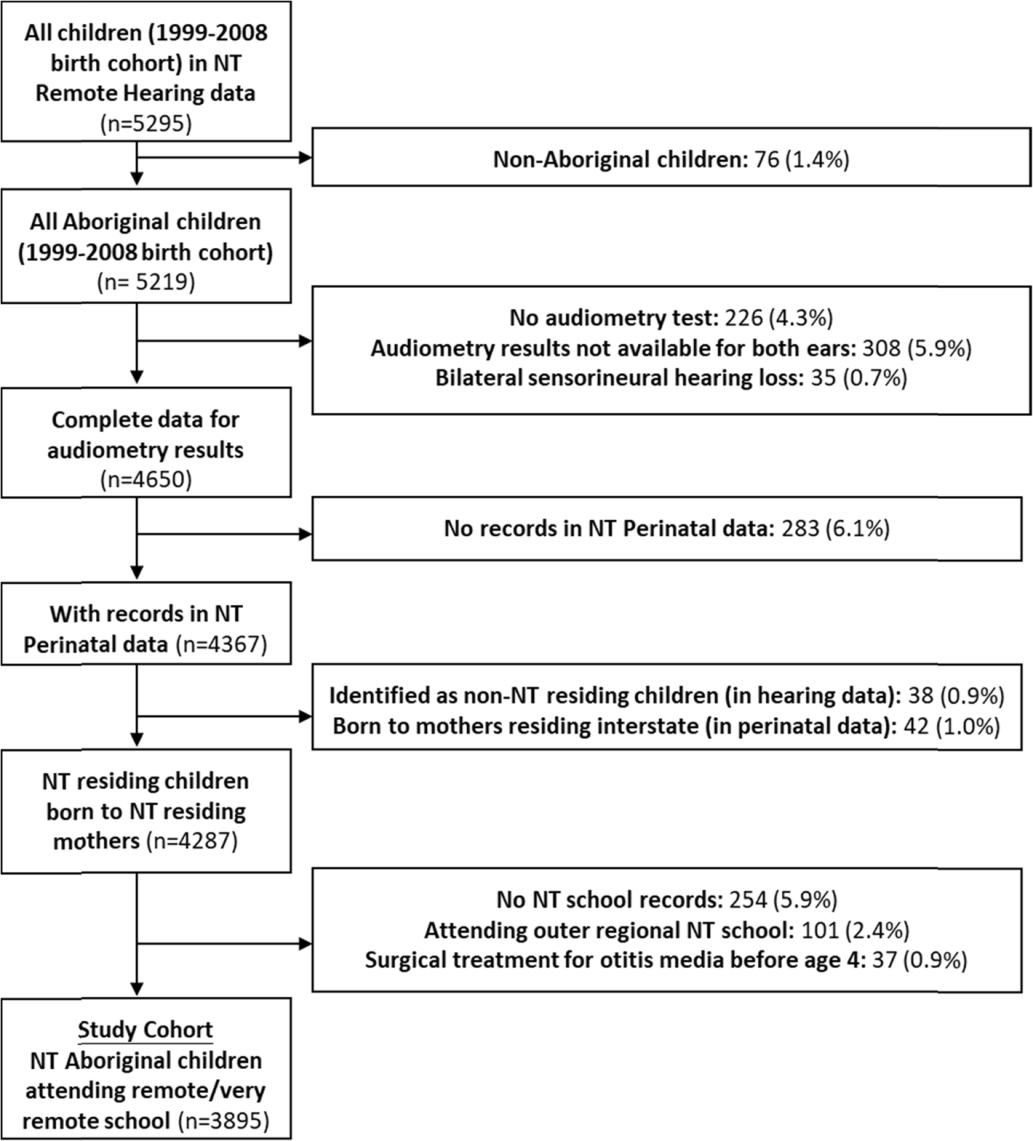
Flow diagram of cohort selection. * Children with a hospital record of otitis media-related surgery, before the age of 4 years, were excluded (37 children) under the premise that early surgery may alter the association between hearing impairment and child maltreatment

This is a retrospective cohort study in which individual-level hearing assessment results were linked to administrative datasets from multiple government agencies. The study cohort was all Aboriginal children; with audiometric assessments (for both ears) recorded in the NT Remote Hearing Assessment dataset, who were born in the NT between 1st January 1999 and 31st December 2008, and who attended school in remote and very remote geographic areas (defined using Accessibility and Remoteness Index of Australia (ARIA+)) [34]. As the focus for this study was on OM-related HI, we excluded all children with sensorineural hearing loss (*n* = 35) from the study (Fig. 1). Records for children in the Remote Hearing Assessment dataset were linked to records from the NT Perinatal Data Register to identify Aboriginal children born to NT residing mothers (*n* = 4287), which was then linked to NT school datasets to identify NT-born Aboriginal children residing in the remote and very remote areas (*n* = 3932). Lastly, the hospital dataset was used to identify and exclude the children with a hospital record of OM-related surgery before the age of 4 years (*n* = 37), under the premise that early surgery may alter the association between HI and child maltreatment. After the cohort selection process, there were 3895 children in our study cohort (Fig. 1).

### Data sources

Data for the study was obtained from the NT child and youth data repository which was established through a collaboration between Menzies School of Health Research and NT Government agencies [35, 36]. Six key administrative datasets were used in the study. The first key dataset was the NT Perinatal Data Register, which is a statutory collection of information for all births in the NT. The second key dataset was the Remote Hearing Assessment dataset, which contains clinical and audiometric records for children assessed by the NT Outreach Hearing Health Program [37]. The third key dataset was the school attendance dataset (from government schools). The fourth key dataset contained results from the annual National Assessment Program – Literacy and Numeracy (NAPLAN) which is undertaken by government and non-government students in Years 3, 5, 7 and 9 [38]. The fifth key dataset contained the statutory records of all contact by children with child protection services. A sixth key dataset, the hospital separations dataset, was used to identify children receiving surgical procedures related to OM. Other datasets in the data repository included other health datasets, mortality records and youth justice records [30, 35].

### Analysis

#### Outcome variables

In the NT, all reports of suspected child maltreatment made to child protection services are recorded as “notifications”. The preferred method of reporting suspected child maltreatment is by telephone to a Central Intake Team which operates a child protection hotline 24 h a day, 7 days a week. Reports from other agencies or reports made to police, or emergency services are also referred to the Central Intake Team. If a notification is consistent with maltreatment, it is referred for investigation, whereby the child protection service “obtains more detailed information about a child who is subject to a notification and staff make an assessment about the harm and the child’s protective needs” [39]. One possible outcome of an investigation is “substantiation”. A “substantiation” refers to an incident reported to child protection services which has been investigated with a conclusion that there is sufficient reason to believe the child has been, is being, or is likely to be abused, neglected, or otherwise harmed. In Australia, child maltreatment is reported as one or more of four types – neglect, physical abuse, sexual abuse (including sexual exploitation) and emotional abuse [39]. Exposure to family violence is reported as emotional abuse. From 1983, all adults in the NT have been required to report any child that they believed at risk of maltreatment, this statutory requirement was reinforced by the *Care and Protection of Children Act 2007 (NT)* which requires all adults to report any child that they believe ‘has suffered or is likely to suffer harm or exploitation’ [40]. In addition, the *Domestic and Family Violence Act 2007 (NT)* introduced mandatory reporting of domestic and family violence, including the reporting by police of children exposed to family violence.

The outcome variables for the study were the first child maltreatment notification and first substantiated child maltreatment notification, for each child, recorded in the child protection dataset. In additional analyses of child maltreatment outcomes by maltreatment type, we used the “primary maltreatment type”. When more than one maltreatment type is recorded for a single event, the primary maltreatment type is the one which is the greatest immediate risk to the child.

#### Explanatory variables

In our study, HI was determined from the first audiometric assessment (for each child) recorded in the Remote Hearing Assessment dataset, under the assumption that the first assessment result was representative of a child’s long-term HI status. This assumption is supported by previous findings that OM in NT Aboriginal children develops early in life [20], is persistent and asymptomatic, and is not diagnosed until an older age due to easier diagnosis [17]. In the NT Outreach Hearing Health Program, hearing assessments were performed using pure tone audiometry with results reported as the average threshold of hearing for the three frequencies: 500 hertz (Hz), 1000 Hz and 2000 Hz [37]. The result for each ear was classified as either normal or one of four levels of hearing loss, namely mild (16–30 dB HL), moderate (31–60 dB HL), severe (61–90 dB HL) and profound (≥ 91 dB HL). Based on these hearing results, we derived the HI variable as a categorical variable containing four mutually exclusive categories as listed below:

- Normal hearing: normal audiometry results in both ears.
- Unilateral hearing loss (UHL): normal in one ear and any degree of hearing loss in the other.
- Mild HI: mild hearing loss in the better hearing ear.
- Moderate or worse HI: moderate or worse hearing loss in the better hearing ear.

In the multivariable analysis, we selected the following additional variables that fell under three categories:

- Child characteristics: sex (being female) and birth order (born as first child)
- Maternal prenatal characteristics: less than seven antenatal visits during pregnancy; a record of the mother drinking alcohol during pregnancy; a record of the mother smoking during pregnancy; a record of maternal diagnosis of a sexually transmitted infection (STI)
- Community characteristics: using the statistical local area (SLA) [41] in which the child first attended school [42].

Information on child and maternal characteristics were obtained from the perinatal dataset and community location was obtained from the school dataset. These variables were selected based on their availability in the NT data repository [30, 35] and evidence, from prior studies, of an association with child maltreatment. A Western Australian study reported a higher risk of child maltreatment among females [43], while an NT study reported greater risks for Aboriginal infants born to mothers with a record of a sexually transmitted infection (STI) during pregnancy, having attended less than seven antenatal visits, having consumed alcohol or having smoked during pregnancy [30]. Birth order was also included in the analysis, based on evidence from previous research that first born children are at greater risk of maltreatment [44]. The variable relating to ‘antenatal care’ is a proxy for maternal access to health care, with the cut-off point of seven antenatal visits based on clinical guidelines for remote NT settings [42]. Other maternal antenatal variables are proxies for the health status or health behaviour of the child’s mother [30, 42]. As previous research has demonstrated geographic variation in child maltreatment rates across the NT [30], we used community location (i.e. SLA [41]) to adjust (as community fixed-effects) for observed and unobserved differences between communities.

#### Aboriginal status

The Aboriginal status variable was derived from a hierarchy based on demonstrated accuracy with health datasets first, followed by child protection, education and youth justice records [42].

### Statistical analysis

Survival analysis was used for various events. For each analysis, the survival time was defined as the time from birth to the occurrence of each event, children who did not experience the event were censored at the earliest of the following: date of death, the last observed date in the linked data or 31 December 2017. The time-scale for the survival analysis was the age of children in years (continuous variable). The Kaplan–Meier estimator was used to calculate the cumulative probability of each outcome at each age, by the four levels of HI. A Cox proportional hazard model was used in the multivariable analysis to examine the association between HI and the first record of child maltreatment. To account for the intra-group (community) correlation standard errors were clustered at the community level. Due to the substantial increase of child protection notifications/substantiations each year, separate baseline hazard rates were estimated for annual birth cohorts in the multivariable analysis, and the cumulative probability of each outcome was reported separately for the 1999–2003 and 2004–2008 birth cohorts. In additional analysis, multivariable analyses were conducted separately for the two cohorts. All statistical analyses were conducted using Stata for Windows, Version 15 (StataCorp 2015).

### Ethical approval

The study was approved by the Human Research Ethic Committee of the NT Department of Health and the Menzies School of Health Research (HREC-2016-2708).

## Results

Table 1 and Additional file 1: Table 1 present the demographic and selected health characteristics for the study cohort of 3895 children (48% girls). The median age for the first hearing assessment was 7.1 years (interquartile range (IQR) =5.5–8.9). The majority of the cohort lived in the most disadvantaged areas [41] (*n* = 3628, 93.1%) and very remote [34] regions (*n* = 3679, 94.5%). Among all children in the study cohort, 56.1% had a record of HI/UHL (*n* = 2168), and for those with a history of contact with child protection services (*n* = 2757), 56.7% had a record of HI/UHL (*n* = 1564).

**Table 1:** Demographic and selected health characteristics of the study cohort (*n* = 3895)

Characteristic	Number	Percent
Demographic characteristics		
Female	1869	48.0
First Child	1304	33.5
Year of Birth		
1999	353	9.1
2000	376	9.7
2001	487	12.5
2002	436	11.2
2003	469	12.0
2004	437	11.2
2005	372	9.6
2006	350	9.0
2007	337	8.7
2008	278	7.1
Maternal and perinatal factors		
Low Birth Weight	526	13.5
Preterm birth	593	15.2
Admitted to special care nursery	1012	26.5^a^
Born to teenage mothers	1270	32.6
Mother attend less than 7 antenatal visits	1334	34.2
Born to mothers with STI	441	11.3
Mother consumed alcohol during pregnancy	372	12.1^b^
Mother smoked during pregnancy	1456	45.4^c^
Hearing assessment		
Normal hearing	1709	43.9
Unilateral hearing loss	740	19.0
Mild hearing impairment (HI)	1078	27.7
Moderate or worse HI	368	9.4
Community level factors		
Living in most disadvantaged areas^d^	3628	93.1
Living in very remote regions^e^	3679	94.5

^a^Denominator only includes those with recorded status of special care nursery (*n* = 3825)

^b^Denominator only includes those with recorded status of maternal alcohol consumption (*n* = 3082)

^c^Denominator only includes those with recorded status of maternal smoking (*n* = 3204)

^d^Index of Relative Socio-Economic Disadvantage (IRSD) from ABS, [19] based on community that the children first attended school

^e^Accessibility and Remoteness Index of Australia (ARIA+) from ABS, [16] based on community that the children first attended school

**Fig. 2:**
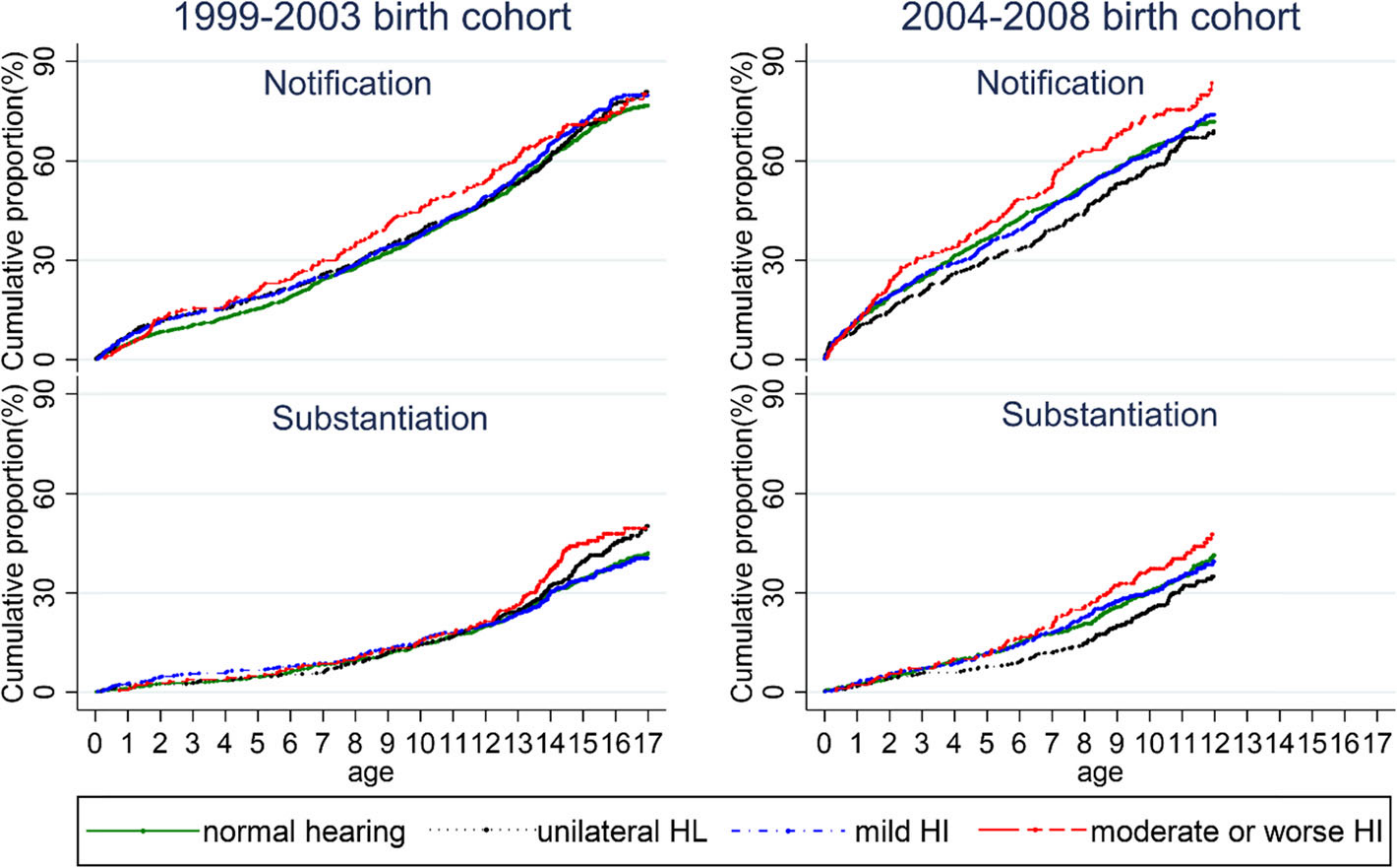
Survival analysis depicting first maltreatment notification and substantiation at different ages, by levels of hearing impairment (i.e. normal hearing, unilateral hearing loss (HL), mild hearing impairment (HI), and moderate or worse HI) for 1999–2003 and 2004–2008 birth cohort

Figure 2 presents the Kaplan-Meier failure curves for first child maltreatment notification and first substantiated cases for the 1999–2003 and 2004–2008 birth cohorts. In the 1999–2003 cohort, by age 12 years, 53.5% of children with moderate or worse HI had at least one maltreatment notification, compared to 47.3% of children with normal hearing. By age 17 years, 80.1% of children (1999–2003 cohort) with moderate or worse HI had at least one maltreatment notification, compared to 76.6% of children with normal hearing. Compared with the 1999–2003 cohort, the 2004–2008 birth cohort had higher cumulative incidence of maltreatment notification and substantiation, by age 12, with 83.4% of children with moderate or worse HI having a maltreatment notification, compared to 71.7% of children with normal hearing. The cumulative incidence of notifications and substantiated cases by different maltreatment types are available in Additional file 1: Tables 2 and 3 respectively.

In multivariable analysis, children with moderate or worse HI had a higher risk of child maltreatment notification (adjusted Hazard Ratios (adjHR:1.16, 95% CI:1.04–1.30) and substantiation (adjHR:1.20, 95% CI: 1.04–1.40) than children with normal hearing (Table 2). Children with moderate or worse HI also had a higher risk of neglect notifications (adjHR:1.17, 95% CI:1.04–1.31) (Additional file 1: Table 4). In the 2004–2008 birth cohort, children with moderate or worse HI had a higher risk of physical abuse substantiations (adjHR:1.47, 95% CI:1.07–2.03) than children with normal hearing. There is also evidence for a higher risk of child maltreatment notifications for girls than boys, and for children with mothers with: a record of an STI, who attended less than 7 antenatal visits, drank alcohol and smoked during pregnancy in the multivariable analysis (Table 2). For first-born children there was no evidence of higher risk of notifications and substantiations for any maltreatment type (Table 2), but some evidence of higher risk of physical abuse notifications, and lower risk of neglect notification and neglect substantiation (Additional file 1: Table 4).

**Table 2:** Multivariable regression results for the association between hearing impairment and risk of child protection notifications and substantiations, 1999–2008 birth cohort, Northern Territory Aboriginal children

	Notification		Substantiation	
	adjHR(95% CI)	p	adjHR(95% CI)	p
Hearing impairment (HI)				
Normal	reference		reference	
Unilateral hearing loss	0.96 (0.88–1.04)	0.290	0.99 (0.90–1.08)	0.808
Mild HI	0.99 (0.88–1.10)	0.820	0.97 (0.82–1.14)	0.693
Moderate or worse HI	1.16 (1.04–1.30)**	0.009	1.20 (1.04–1.40)*	0.015
Gender				
Male	reference		reference	
Female	1.09 (1.01–1.17)*	0.021	1.09 (0.94–1.26)	0.244
First Child				
No	reference		reference	
Yes	0.93 (0.85–1.02)	0.141	0.95 (0.87–1.04)	0.246
Born to mother with STI				
No	reference		reference	
Yes	1.24 (1.11–1.40)***	< 0.001	1.25 (1.07–1.45)**	0.004
Mother attend less than 7 antenatal visits				
No	reference		reference	
Yes	1.03 (1.00–1.06)*	0.030	1.02 (0.91–1.14)	0.792
Mother consumed alcohol during pregnancy				
No	reference		reference	
Yes	1.34 (1.15–1.55)***	< 0.001	1.39 (1.16–1.67)***	< 0.001
Not stated/missing	1.13 (0.99–1.29)	0.072	1.00 (0.84–1.19)	0.975
Mother smoked during pregnancy				
No	reference		reference	
Yes	1.22 (1.09–1.38)**	0.001	1.14 (0.99–1.30)	0.061
Not stated/missing	1.12 (0.99–1.27)	0.076	1.13 (0.96–1.32)	0.135

1. adjHR:adjusted HR.

2. The results are adjusted for community fixed effect

3. *: *p* values < 0.05; **: *p* values < 0.01; ***: *p* values < 0.001

## Discussion

Our study confirms the high prevalence of preventable HI among Aboriginal children in remote communities of the NT and provides the first empirical evidence of an association between HI and an increased risk of child maltreatment in this setting, with higher rates of both notifications and substantiated episodes of maltreatment among Aboriginal children with moderate (or worse) HI. There are also higher risks for neglect notifications (1999–2008 birth cohort) and physical abuse substantiations (2004–2008 birth cohort) amongst children with moderate (or worse) HI. These findings are consistent with previous studies which have reported higher risk for neglect and physical abuse for children with HI [3, 5, 45]. These same studies also found a higher risk of emotional abuse amongst children with HI which was not demonstrated in our study. This may be a reflection of the particular reporting requirements in the NT over the last decade including, for example, the statutory requirement for police to report all children, exposed to domestic violence [46], which has contributed to a substantial increase in reports of emotional abuse, independent of the underlying risks for individual children [29]. It is also important to note that previous research on the association between HI and child maltreatment has been undertaken in setting in which both conditions have relatively low prevalence; further research is warranted to better understand HI-maltreatment link in the NT remote settings.

Our study has several significant implications for service providers. Firstly, the findings highlight the critical role of health practitioners in preventing maltreatment arising from hearing-related communication problems—when health practitioners identify ear disease or hearing loss, they are in a strategic position to provide information to families on effective treatment options (whether medical, audiological or surgical) and the likely communication difficulties and how to avoid them [47]. Secondly, the high prevalence of HI among children with child maltreatment notifications reported in our study highlight the need for training child protection workers in ‘hearing loss responsive communication strategies’ [48, 49], which was recommended in a 2017 Aboriginal health report for all workers engaged with Aboriginal people [50]. Thirdly, screening for ear disease and HI needs to be incorporated into the assessment and treatment of children by child protection services, through integration with the health services, in order to provide adequate support to children, families and foster carers, which has the potential to reduce maltreatment recurrence as well as improve treatment and recovery [49]. The provision of comprehensive health screening (including hearing tests) to children in out-of-home care (or children referred by the Community Services) by the Child Protection Units in the Sydney Children’s Hospitals Network provides an example of multi-disciplinary teams (with social workers, specialist medical and psycho-social health professionals) working within an interagency framework [51–54]. In addition, many preventive health programs and initiatives can contribute to the prevention of CM by detecting OM and/or HI early, such as the Healthy Under 5 Kids program, which includes ear assessment and targets all Aboriginal children in remote communities [55]. Later screening activities and interventions are also important, for example, ear and hearing screening on entry into preschool or primary school [56]. The interventions may have longer term benefits, including reduction in the risk of children progressing from child protection services to the youth justice system, a setting in which there are also reports of high levels of HI among Aboriginal youth [57]. Fourthly, the use of assistive listening devices for victims of maltreatment, as well as perpetrators, who may also have communication and psychosocial difficulties related to HI [58], is important in providing adequate communication with police, child protection case workers, legal representatives, medical and counselling services [48]. Fifthly, our study reinforces the need to recognise cultural and linguistic diversity in strategies that respond to learning and communication difficulties related to HI [59]. As an example, in this setting, it is important to recognize the utility of Aboriginal sign languages (“hand talk”) for Aboriginal children with HI in addition to more widely used strategies to assist school learning such as sound systems and improved classroom acoustic management. Sixthly, the high rates of preventable HI and child maltreatment reports in remote communities highlight the importance of interagency collaboration, including between health, education and child protection services and place-based strategies, in partnership with communities, “built on the principles of mutual respect, shared commitment, shared responsibility and good faith” [31]. While our finding of the link between HI and physical abuse might help to inform how communication barriers resulting from hearing impairment of children can contribute to use of physical discipline by carers, our finding of the link between HI and neglect (mostly associated with poverty) suggests the greater importance of tackling the common social determinants (such as poverty and overcrowding) of preventable HI and child maltreatment at a population-level, which is only possible through a whole-of-community approach and inter-agency collaboration.

Our study has several limitations. Firstly, not all Aboriginal children accessed the ear health outreach service; it has been reported that about 18% of NT Aboriginal people, aged under 21, received the outreach audiology services [37]. In addition, given that the outreach services target children with high needs, the children who accessed these services are not a random sample of NT Aboriginal children. Care is required if generalising the results to all NT Aboriginal children. The new ‘Hearing for Learning’ program is expected to increase the proportion of young NT Aboriginal children receiving regular ear and hearing assessment and may lead to more universal collection of hearing assessment data and better population coverage, resulting in a more representative data [60]. Secondly, the availability and timing of the hearing assessment [37] made it necessary to use each child’s first audiometry result for analysis, under the assumption that the result was indicative of long-term hearing status of a child. As the severity of HI may change with time, this approach may have resulted in some misclassification. The misclassification is likely to result in an underestimation of the strength of the association between HI and child maltreatment as children with HI in early childhood might have higher risk of child maltreatment even with a later improvement in hearing status. Thirdly, although prior literature indicated that risk factors such as deprivation of language (resulting from HI) may increase the risk of child maltreatment, this study was unable to ascertain causality nor the direction of the association between HI and maltreatment. It is possible, for example, that neglect in early life may have contributed to OM being untreated. Fourthly, the rapid increase in reporting of child maltreatment between 1999 and 2017 [30] means that there was underestimation of the levels of child maltreatment, particularly for earlier birth cohorts. Fifthly, although we have adjusted for selected maternal and community factors in the multivariable model, there are other factors, including child intellectual disability, parental socio-economic status and maternal mental health [43] that may be important confounders but which were not available for this study.

## Conclusion

Our study provides evidence for an association between preventable HI and child maltreatment for Aboriginal children living in NT remote communities. The high rates of both HI and child maltreatment in remote communities highlight the urgent need for prevention strategies through raised community awareness and improved response of the health, education, welfare, child protection and justice services [48]. To achieve such a goal, effective information-sharing between services is a critical first step in informing a coordinated approach to service delivery to meet the needs of NT children [31, 61, 62].

## Supplementary information

**Additional file 1: Table 1.** Demographic and selected health characteristics of the study cohort, by levels of hearing impairment (HI). **Table 2.** Cumulative incidence (95% confidence interval) of the first child maltreatment notification or substantiation, by type, for NT-born Aboriginal children in NT Remote Hearing Dataset (1999–2003 and 2004–2008 birth cohorts) at age 12 years, by levels of hearing impairment (HI). **Table 3.** Cumulative incidence (95% confidence interval) of first child maltreatment notification or substantiation by type, for NT-born Aboriginal children in NT Remote Hearing Dataset (1999–2003 birth cohort) at age 17 years, by levels of hearing impairment (HI). **Table 4.** Multivariable regression (fixed effect model) for child maltreatment notifications and substantiations, by type, for levels of hearing impairment (HI), NT-born Aboriginal children, 1999–2008 birth cohort, Northern Territory.

## Data Availability

The study datasets contain sensitive personal information and are held on a secure cloud-based server with restricted access. Access requires the approval of the ethics committee and data custodians.

## Abbreviations

ABS: Australian Bureau of Statistics
adjHR: Adjusted hazard ratios
ARIA+: Accessibility and Remoteness Index of Australia
HI: Hearing impairment
NAPLAN: National Assessment Program – Literacy and Numeracy
NT: Northern Territory
OM: Otitis media
SLA: Statistical local area
STI: Sexually transmitted infection
UHL: Unilateral hearing loss.
