# Supplementary material for: The link between hearing impairment and child maltreatment among Aboriginal children in the Northern Territory of Australia: is there an opportunity for a public health approach in child protection?
Source: BMC Public Health. 2020 Apr 6;20:449. doi: 10.1186/s12889-020-8456-8 (PMC7132974; doi:10.1186/s12889-020-8456-8)
Supplement: Supplementary file 1 — Additional file 1: Table 1. Demographic and selected health characteristics of the study cohort, by levels of hearing impairment (HI). Table 2. Cumulative incidence (95% confidence interval) of the first child maltreatment notification or substantiation, by type, for NT-born Aboriginal children in NT Remote Hearing Dataset (1999–2003 and 2004–2008 birth cohorts) at age 12 years, by levels of hearing impairment (HI). Table 3. Cumulative incidence (95% confidence interval) of first child maltreatment notification or substantiation by type, for NT-born Aboriginal children in NT Remote Hearing Dataset (1999–2003 birth cohort) at age 17 years, by levels of hearing impairment (HI). Table 4. Multivariable regression (fixed effect model) for child maltreatment notifications and substantiations, by type, for levels of hearing impairment (HI), NT-born Aboriginal children, 1999–2008 birth cohort, Northern Territory. [file 12889_2020_8456_MOESM1_ESM.docx]

# **Appendix**

Appendix Table 1: Demographic and selected health characteristics of the study cohort, by levels of hearing impairment (HI).

| Characteristic | Levels of hearing impairment | | | | |
| --- | --- | --- | --- | --- | --- |
|  | Normal hearing (n=1709) | Unilateral hearing loss (n=740) | Mild HI (n=1078) | Moderate or worse HI (n=368) |  |
| Demographic characteristics | |  |  |  |  |
| Female | 812(47.5%) | 361(48.8%) | 506(46.9%) | 190(51.6%) |  |
| First Child | 575(33.6%) | 255(34.5%) | 342(31.7%) | 132(35.9%) |  |
| Maternal and perinatal factors |  |  |  |  |  |
| Low Birth Weight | 201(11.8%) | 98(13.2%) | 169(15.7%) | 58(15.8%) |  |
| Preterm birth | 230(13.5%) | 103(13.9%) | 193(17.9%) | 67(18.2%) |  |
| Admitted to special care nursery ^a^ | 418(24.5%) | 172(23.2%) | 319(29.6%) | 103(28.0%) |  |
| Born to teenage mothers | 534(31.2%) | 255(34.5%) | 353(32.7%) | 128(34.8%) |  |
| Mother attend less than 7 antenatal visits | 582(34.1%) | 235(31.8%) | 403(37.4%) | 114(31.0%) |  |
| Born to mothers with STI | 195(11.4%) | 82(11.1%) | 114(10.6%) | 50(13.6%) |  |
| Mother consumed alcohol during pregnancy ^b^ | 158(11.5%) | 73(12.3%) | 109(13.2%) | 32(10.9%) |  |
| Mother smoked during pregnancy ^c^ | 619(43.7%) | 319(50.6%) | 374(43.6%) | 144(47.8%) |  |
| Age of first hearing assessment |  |  |  |  |  |
| First quartile (25^th^ percentile) | 6.2 | 5.6 | 5.1 | 4.7 |  |
| Median (50^th^ percentile) | 7.8 | 7.1 | 6.5 | 6.2 |  |
| Third quartile (75^th^ percentile) | 9.4 | 8.8 | 8.0 | 8.0 |  |
| Community level factors | 6.2 | 5.6 | 5.1 | 4.7 |  |
| Living in most disadvantaged areas^d^ | 1565(91.6%) | 701(94.7%) | 1012(93.9%) | 350(95.1%) |  |
| Living in very remote regions^e^ | 1604(93.9%) | 700(94.6%) | 1027(95.3%) | 348(94.6%) |  |
| Year of birth |  |  |  |  |  |
| 1999-2003 | 1004(58.7%) | 402(54.3%) | 528(49.0%) | 187(50.8%) |  |
| 2004-2008 | 705(41.3%) | 338(45.7%) | 550(51.0%) | 181(49.2%) |  |

1. Denominator only includes those with recorded status of special care nursery (n=3825).
2. Denominator (for proportion,%) only includes those with recorded status of maternal alcohol consumption (n=3082).
3. Denominator (for proportion,%) only includes those with recorded status of maternal smoking (n=3204).
4. Index of Relative Socio-Economic Disadvantage (IRSD) from ABS,^19^ based on community that the children first attended school.
5. Accessibility and Remoteness Index of Australia (ARIA+) from ABS,^16^ based on community that the children first attended school.

Appendix Table 2: Cumulative incidence (95% confidence interval) of the first child maltreatment notification or substantiation, by type, for NT-born Aboriginal children in NT Remote Hearing Dataset (1999-2003 and 2004-2008 birth cohorts) at age 12 years, by levels of hearing impairment (HI).

|  | Abuse type | 1999-2003 Birth Cohort | | | | 2004-2008 Birth Cohort | | | |
| --- | --- | --- | --- | --- | --- | --- | --- | --- | --- |
|  |  | Normal hearing | Unilateral HL | Mild HI | Moderate HI | Normal hearing | Unilateral HL | Mild HI | Moderate HI |
| Notification | Any | 47.3(44.3-50.5) | 47.3(42.6-52.3) | 49.1(44.9-53.5) | 53.5(46.5-60.8) | 71.7(68.0-75.4) | 68.9(63.2-74.5) | 73.9(69.6-78.0) | 83.4(75.7-89.8) |
|  | Neglect | 30.7(28.0-33.7) | 30.6(26.4-35.4) | 28.9(25.2-32.9) | 38.0(31.4-45.3) | 55.2(51.0-59.5) | 50.7(44.7-57.0) | 54.7(50.1-59.4) | 65.8(57.7-73.8) |
|  | Physical | 15.1(13.0-17.5) | 19.9(16.4-24.2) | 18.8(15.7-22.4) | 23.0(17.6-29.7) | 37.9(33.9-42.2) | 32.5(27.4-38.4) | 37.9(33.4-42.7) | 41.9(34.3-50.4) |
|  | Emotional | 13.4(11.4-15.6) | 13.5(10.5-17.2) | 12.2(9.7-15.3) | 15.5(11.0-21.5) | 41.2(37.3-45.5) | 37.0(31.2-43.4) | 42.0(37.3-47.0) | 44.6(36.9-53.1) |
| Substantiation | Any | 20.0(17.6-22.6) | 20.2(16.6-24.5) | 20.1(16.9-23.8) | 21.4(16.2-28.0) | 41.4(37.2-45.8) | 35.0(29.4-41.3) | 39.5(35.0-44.3) | 47.7(39.8-56.3) |
|  | Neglect | 12.1(10.2-14.2) | 12.2(9.4-15.8) | 11.2(8.8-14.2) | 13.4(9.2-19.1) | 26.7(23.1-30.7) | 23.0(18.1-29.1) | 27.6(23.5-32.2) | 30.2(23.2-38.7) |
|  | Physical | 4.3(3.2-5.7) | 5.5(3.6-8.2) | 5.1(3.5-7.4) | 5.9(3.3-10.4) | 13.0(10.4-16.3) | 13.5(9.8-18.4) | 12.5(9.6-16.1) | 18.5(13.0-25.9) |
|  | Emotional | 1.2(0.7-2.1) | 0.7(0.2-2.3) | 1.1(0.5-2.5) | 0 | 5.7(4.0-8.2) | 6.6(4.1-10.7) | 3.6(2.2-6.0) | 4.2(1.9-9.3) |

Appendix Table 3: Cumulative incidence (95% confidence interval) of first child maltreatment notification or substantiation by type, for NT-born Aboriginal children in NT Remote Hearing Dataset (1999-2003 birth cohort) at age 17 years, by levels of hearing impairment (HI).

|  | Abuse type | Normal hearing | Unilateral HL | Mild HI | Moderate HI |
| --- | --- | --- | --- | --- | --- |
| Notification | Any | 76.6(73.4-79.7) | 80.8(75.5-85.6) | 79.7(75.3-83.8) | 80.1(72.5-86.8) |
|  | Neglect | 65.8(62.1-69.5) | 69.1(63.2-74.9) | 63.0(57.7-68.3) | 65.4(57.4-73.3) |
|  | Physical | 32.3(28.9-36.1) | 40.7(34.6-47.5) | 37.0(31.7-42.8) | 36.0(28.6-44.6) |
|  | Emotional | 31.5(28.3-35.0) | 38.6(32.7-45.2) | 33.6(28.6-39.2) | 41.6(32.5-52.0) |
| Substantiation | Any | 42.0(38.4-45.7) | 50.2(44.0-56.7) | 40.5(35.6-45.8) | 49.5(41.7-58.0) |
|  | Neglect | 30.6(27.2-34.3) | 34.7(29.2-40.9) | 25.5(21.3-30.3) | 32.2(25.5-40.2) |
|  | Physical | 11.1(8.8-13.9) | 12.9(9.1-18.0) | 9.9(7.2-13.6) | 10.6(6.7-16.5) |
|  | Emotional | 3.2(2.1-4.8) | 4.2(2.1-8.3) | 2.8(1.5-5.0) | 3.6(1.2-10.0) |

Appendix Table 4: Multivariable regression (fixed effect model) for child maltreatment notifications and substantiations, by type, for levels of hearing impairment (HI), NT-born Aboriginal children, 1999-2008 birth cohort, Northern Territory

|  | Notifications | | | | | | Substantiation | | | | | |
| --- | --- | --- | --- | --- | --- | --- | --- | --- | --- | --- | --- | --- |
|  | Neglect | | Physical | | Emotional | | Neglect | | Physical | | Emotional | |
|  | adjHR (95% CI) | p | adjHR (95% CI) | p | adjHR (95% CI) | p | adjHR (95% CI) | p | adjHR (95% CI) | p | adjHR (95% CI) | p |
| Hearing impairment | | | | | | | | | | | | |
| Normal | 1.00 | | 1.00 | | 1.00 | | 1.00 | | 1.00 | | 1.00 | |
| Unilateral hearing loss | 0.95(0.86-1.05) | 0.309 | 1.00(0.84-1.20) | 0.967 | 0.91(0.81-1.02) | 0.103 | 0.99(0.87-1.13) | 0.915 | 1.00(0.80-1.25) | 0.992 | 1.24(0.75-2.03) | 0.405 |
| Mild HI | 0.94(0.82-1.07) | 0.344 | 1.01(0.85-1.19) | 0.945 | 0.88(0.77-1.00) | 0.057 | 0.96(0.83-1.11) | 0.575 | 0.91(0.68-1.23) | 0.540 | 0.65(0.36-1.18) | 0.160 |
| Moderate or worse HI | 1.17(1.04-1.31)** | 0.007 | 1.09(0.88-1.35) | 0.416 | 0.97(0.74-1.29) | 0.859 | 1.12(0.90-1.40) | 0.320 | 1.27(0.95-1.71) | 0.105 | 0.72(0.25-2.03) | 0.533 |
| Gender | | | | | | | | | | | | |
| Male | 1.00 | | 1.00 | | 1.00 | | 1.00 | | 1.00 | | 1.00 | |
| Female | 1.01(0.95-1.07) | 0.852 | 1.09(1.01-1.18)* | 0.022 | 1.15(1.03-1.29)* | 0.013 | 1.07(0.88-1.31) | 0.480 | 0.93(0.79-1.10) | 0.418 | 1.33(0.96-1.83) | 0.088 |
| First Child | | | | | | | | | | | | |
| No | 1.00 | | 1.00 | | 1.00 | | 1.00 | | 1.00 | | 1.00 | |
| Yes | 0.86(0.79-0.94)** | 0.001 | 1.12(1.00-1.26)* | 0.049 | 1.07(0.92-1.25) | 0.358 | 0.87(0.76-0.99)* | 0.034 | 1.08(0.87-1.33) | 0.482 | 1.24(0.90-1.71) | 0.182 |
| Born to mother with STI | | | | | | | | | | | | |
| No | 1.00 | | 1.00 | | 1.00 | | 1.00 | | 1.00 | | 1.00 | |
| Yes | 1.27(1.12-1.45)*** | <0.001 | 1.00(0.80-1.25) | 0.969 | 1.13(0.89-1.42) | 0.313 | 1.31(1.09-1.57)** | 0.003 | 0.97(0.67-1.41) | 0.873 | 1.45(1.13-1.86)** | 0.003 |
| Mother attend less than 7 antenatal visits | | | | | | | | | | | | |
| No | 1.00 | | 1.00 | | 1.00 | | 1.00 | | 1.00 | | 1.00 | |
| Yes | 1.09(1.02-1.16)* | 0.015 | 0.98(0.91-1.06) | 0.690 | 0.96(0.85-1.08) | 0.510 | 1.12(0.96-1.30) | 0.145 | 1.03(0.84-1.26) | 0.776 | 0.74(0.55-1.01) | 0.058 |
| Mother drank alcohol during pregnancy | | | | | | | | | | | | |
| No | 1.00 | | 1.00 | | 1.00 | | 1.00 | | 1.00 | | 1.00 | |
| Yes | 1.37(1.22-1.54)*** | <0.001 | 1.25(1.00-1.58) | 0.055 | 0.99(0.74-1.32) | 0.929 | 1.25(1.03-1.52)* | 0.023 | 1.77(1.25-2.50)** | 0.001 | 1.14(0.65-2.00) | 0.647 |
| Not stated/missing | 1.14(0.94-1.39) | 0.190 | 1.17(0.87-1.57) | 0.291 | 0.98(0.73-1.31) | 0.875 | 1.18(0.94-1.48) | 0.158 | 1.39(1.06-1.81)* | 0.016 | 0.58(0.27-1.25) | 0.165 |
| Mother smoked during pregnancy | | | | | | | | | | | | |
| No | 1.00 | | 1.00 | | 1.00 | | 1.00 | | 1.00 | | 1.00 | |
| Yes | 1.35(1.11-1.66)** | 0.003 | 1.31(1.07-1.61)** | 0.009 | 1.23(1.11-1.37)*** | <0.001 | 1.24(0.96-1.58) | 0.096 | 1.17(0.84-1.63) | 0.360 | 1.31(1.01-1.71)* | 0.042 |
| Not stated/missing | 1.12(0.91-1.38) | 0.273 | 1.12(0.89-1.40) | 0.352 | 1.25(0.94-1.67) | 0.129 | 1.04(0.79-1.38) | 0.772 | 1.04(0.80-1.34) | 0.779 | 2.30(1.23-4.30)** | 0.009 |

Notes:

1. adjHR:adjusted HR.

2. The results are adjusted for community fixed effect

3. *: p values <0.05; **: p values <0.01; ***: p values <0.001.
